# Neoadjuvant immunotherapy combined with chemotherapy significantly improved patients’ overall survival when compared with neoadjuvant chemotherapy in non-small cell lung cancer: A cohort study

**DOI:** 10.3389/fonc.2022.1022123

**Published:** 2022-10-24

**Authors:** Fuqiang Dai, Xiaoli Wu, Xintian Wang, Kunkun Li, Yingjian Wang, Cheng Shen, Jinghai Zhou, Huijun Niu, Bo Deng, Qunyou Tan, Ruwen Wang, Wei Guo

**Affiliations:** Department of Thoracic Surgery, Daping Hospital, Army Medical University, Chongqing, China

**Keywords:** neoadjuvant immunotherapy, lung resection, prognosis, non-small cell lung cancer, neoadjuvant chemotherapy

## Abstract

**Background:**

Programmed death-1 (PD-1)/programmed death ligand-1 (PD-L1) inhibitors displayed considerable advantages in neoadjuvant therapy of non-small cell lung cancer (NSCLC), but the specific application of neoadjuvant immunotherapy has not been well determined, and the long-term prognostic data of neoadjuvant immunochemotherapy combined with surgical resection of NSCLC remains limited. In this study, we intended to assess the efficacy of the neoadjuvant therapy of the PD-1 inhibitor and long-term prognosis in patients with resectable NSCLC.

**Methods:**

We retrospectively analyzed NSCLC surgical patients treated with neoadjuvant therapy in our hospital, and divided them into a neoadjuvant chemotherapy group and a neoadjuvant immunotherapy combined with chemotherapy group. The propensity score matching method was used to evaluate the effectiveness of immunotherapy combined with chemotherapy in the treatment of resectable lung cancer, and the long-term prognosis of these two groups was compared.

**Results:**

A total of 62 cases were enrolled, including 20 patients (20/62, 32.26%) in the immunotherapy group and 42 patients (42/62, 67.74%) in the chemotherapy group. The clinical baseline data of these two groups were balanced. In the immunotherapy group, all patients had tumor regression in imaging finding (tumor regression ratio: 11.88% - 75.00%). In the chemotherapy group, 30 patients had tumor regression (tumor regression ratio: 2.70% - 58.97%). The R0 removal rates of cancers were comparable between the immunotherapy group and chemotherapy group (19/20, 95.00% vs. 39/42, 92.86%, P=1.000). The two groups were balanced in complete minimally invasive surgery, pneumonectomy, operative duration, blood loss, postoperative complications, and hospital stay. The immunotherapy group had more sleeve resection (36.84% vs. 10.26%, p=0.039) including bronchial sleeve and vascular sleeve, higher pathological complete response (pCR) rate (57.89% vs. 5.13%, P<0.001) and major pathologic response (MPR) rate (78.95% vs. 10.26%, P<0.001). There were no differences in survival curves for: smoker and non-smoker, squamous cell carcinoma and adenocarcinoma, or right lung cancer and left lung cancer. Moreover, patients who achieved MPR (including pCR) had significantly better overall survival (OS) and disease-free survival (DFS). Patients in immunotherapy group had significantly better OS and longer DFS than those in chemotherapy group.

**Conclusions:**

In conclusion, neoadjuvant immunotherapy combined with chemotherapy can provide better OS and DFS and improving pCR and MPR rates by shrinking tumors.

This study has been registered in the Chinese Clinical Trial Registry, number ChiCTR2200060433. http://www.chictr.org.cn/edit.aspx?pid=170157&htm=4.

## Introduction

Lung cancer is one of the most common cancers with extremely high morbidity and mortality rates worldwide (1, 2). Surgical resection is the main strategy for the treatment of early-stage non-small cell lung cancer (NSCLC), which has a high cure rate. However, patients with NSCLC have a poor prognosis after surgery with 5-year survival rates at approximately 50% for stage II and 20% for stage III, even if the tumor is completely removed (3). This poor prognosis may be a result of tumor metastasis or recurrence caused by residual tumor cells, tumor micro metastases, or circulating tumor cells (CTC) and circulating tumor DNA (ctDNA). Even neoadjuvant or adjuvant radiotherapy or chemotherapy can only improve the 5-year survival rate by 5%, which is relatively limited (4, 5). Therefore, novel neoadjuvant therapeutic strategies are urgently needed to reduce the risk of recurrence and further prolong the survival of patients with NSCLC.

Our understanding of the role of the immune system in the regulation of tumor development has significantly increased in recent years, which makes the promise of immunotherapy a revolution in the treatment of cancer. Immune checkpoints have been showed to regulate the immune response during tumor development (6, 7). Immune checkpoint inhibitors (ICIs) targeting programmed cell death-1 (PD-1) or its ligand (PD-L1) have achieved significant improvements in clinical adjuvant therapy for esophageal/esophagogastric junction carcinoma (8), bladder cancer (9), melanoma (10), and lung cancer (11). ICIs have become an important treatment for advanced non-small cell lung cancer, which can greatly improve the 5-year overall survival (OS) of patients (11, 12). In addition, ICIs also show considerable advantages in short-term results of NSCLC neoadjuvant therapy, such as safety, tolerability, and major pathological response (MPR), when compared with conventional neoadjuvant therapy (13–15). Neoadjuvant immunotherapy could adequately activate the immune response and may remove residual lesions or small metastases (16). However, the application of neoadjuvant immunotherapy has not been well established, and long-term prognostic data of neoadjuvant immunochemotherapy combined with surgical resection of NSCLC remains limited.

Therefore, in the current study, we retrospectively analyzed patients of NSCLC undergoing surgery after neoadjuvant therapy, which were then divided into a neoadjuvant chemotherapy group (Che. group) and a neoadjuvant immunotherapy combined chemotherapy group (Imm. group). The aims of this study were to evaluate the efficacy of neoadjuvant immunotherapy combined with chemotherapy in the treatment of resectable NSCLC, and to compare the long-term prognosis between the two groups. We present the following article in accordance with the STROBE reporting checklist.

## Methods

### Patients

The database of the Army Medical Center of Chinese People’s Liberation Army (PLA) (Daping Hospital) was searched retrospectively from January 2017 to October 2021. This study was reviewed and approved by the ethics committee of the hospital (Ethics Committee of Army Medical Center of PLA, approval number: 2021-273). Individual consent for this retrospective analysis was waived. The study was performed in accordance with the Declaration of Helsinki. Patients who met the following criteria were included: (I) males or females aged 20-75 years; (II) initially diagnosed as NSCLC (clinical stage IB - IIIB) and treatment-naive; (III) resectable lung cancer at the first multidisciplinary diagnosis and treatment (MDT) assessment; (IV) Karnofsky performance status (KPS) ≥ 80, and tolerant to neoadjuvant therapy; (V) receiving neoadjuvant therapy prior to resection; (6) no targeted gene mutations in genetic testing and PD-L1 expression positive in immunohistochemical staining. Patients who met the following criteria were excluded: (I) poor cardiopulmonary function and intolerance of surgery due to cardiopulmonary or other organ dysfunction; (II) tumor progression to unresectable or distant metastasis after neoadjuvant therapy at the second MDT assessment; (III) patients with autoimmune diseases or using immunosuppressive drugs over a long-term; (IV) refusal to undergo follow-up. Data regarding age, sex, smoking status, predicted percentage of the forced expiratory volume (FEV1%), tumor size, tumor location, pathologic type of tumor, clinical stage of tumor, type of operation, operation time, blood loss during operation, postoperative complications, and hospital stay were collected and analyzed. The UICC/AJCC TNM Staging System Eighth Edition for NSCLC was used in this study to evaluate the tumor (17).

### Treatment options

**Figure 1:**
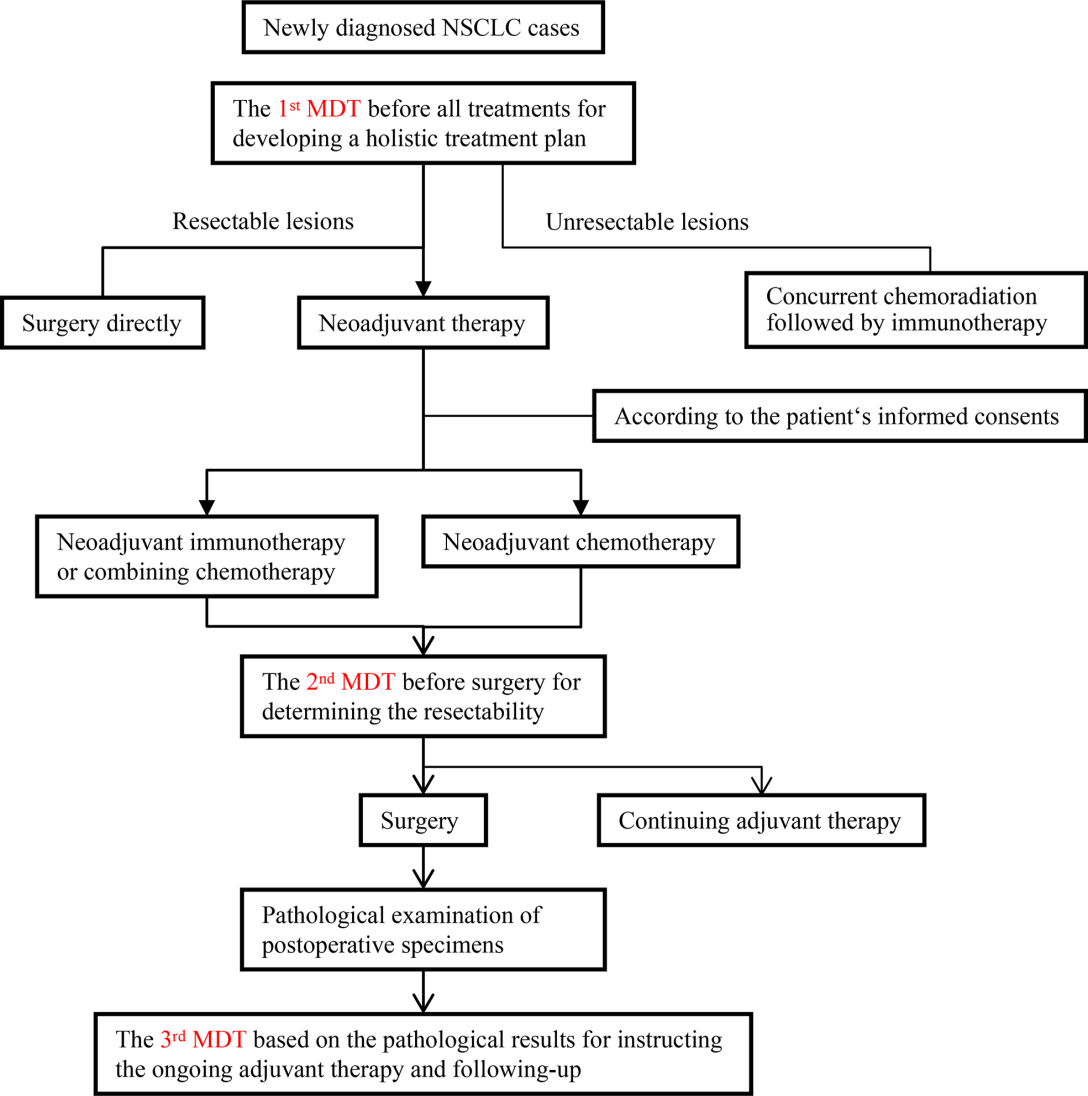
Flowchart summarizing the three multidisciplinary diagnosis and treatments (MDTs) and process of managing patients. NSCLC, non-small cell lung cancer.

All treatment protocols of patients were conducted by MDT. There were 3 times MDTs: the first for the initial assessment, the second for the post-neoadjuvant therapy assessment, and the third for the adjuvant treatment and follow-up assessment (**Figure 1**).

Contrast-enhanced computed tomography (CT) of the chest, ^18^F-fluorodeoxyglucose positron emission tomography/computed tomography (18F-FDG PET-CT), cardiopulmonary function, blood test, and fiberoptic bronchoscopy or percutaneous lung puncture for obtaining pathological results were performed. Immunohistochemistry stains were conducted to detect the expression level of PD-L1 protein in tumor cells. The expression level of PD-L1 was indicated by the percentage of stained cells, which was <1% defined as negative expression and ≥1% defined as positive expression.

Two cycles of neoadjuvant chemotherapy or neoadjuvant immunotherapy combined with chemotherapy were performed. The neoadjuvant chemotherapy regimen was pemetrexed (500 mg/m^2^, D1) + nedaplatin (75 mg/m^2^, D1) for adenocarcinoma and paclitaxel liposome (175 mg/m^2^, D1) + nedaplatin (75 mg/m^2^, D1) for squamous carcinoma (the use of chemotherapy drugs was based on NCCN guidelines and Chinese Lung cancer Diagnosis and Treatment Guidelines (18)). The neoadjuvant immunotherapy was PD-1 inhibitors, including tislelizumab, nivolumab, pembrolizumab, sintilimab. In three to six weeks after completion of these treatments, chest CT and PET-CT are performed again to assess changes of the tumors.

Surgical procedures included video-assisted thoracoscopic surgery (VATS) or thoracotomy, lobectomy, bronchial or vascular sleeve resection, and mediastinal lymph node dissection. All patients were sent back to the thoracic surgery unit after surgery (after the operation, patients were required to stay for a short time in the recovery room until recovering from anesthesia). The patients were encouraged to cough and expectorate to promote drainage and pulmonary re-expansion and were instructed for early activities. Patients whose 24-hour chest drainage volume was less than 200 mL, had no pneumothorax or residual space on chest radiograph, and had no air leakage from the chest tube underwent chest tube removal.

The patients were admitted 1 month after surgery for the third MDT and further therapy according to the guidelines (12, 19) and our MDT recommendations. Patients in the neoadjuvant chemotherapy group received four cycles of adjuvant chemotherapy with the same regimen as before surgery. Patients in the neoadjuvant immunotherapy group received four cycles of adjuvant chemotherapy and six cycles of adjuvant immunotherapy with the same protocol as before surgery. During the follow-up, once tumor recurrence was found, the tolerance and tumor status of patients needed to be assessed by general examination, and the tissue of recurrent lesion should be obtained as far as possible for pathological and genetic testing. Through MDT assessment, individualized therapy plans were set out and implemented after patient’s informed consent.

The postoperative overall survival (OS) was defined as the time from primary tumor resection (surgical date) to last follow-up or death. Disease-free survival (DFS) was defined as the time from the surgical date to the diagnosis of recurrence/metastasis or the last follow-up.

### Tumor evaluation

The tumors were evaluated twice: imaging evaluation after 2 cycles of neoadjuvant therapy and postoperative pathological evaluation.

The imaging response of tumors to neoadjuvant therapy was reviewed centrally by two radiologists according to the Response Evaluation Criteria in Solid Tumor 1.1 (iRECIST Criteria 1.1) (20). To assess changes in primary tumor after neoadjuvant therapy, we recorded the tumor diameter.

We reextracted paraffin-embedded postoperative specimens previously processed by the pathology department and scored the percentage of residual tumor cells by two trained pathologists. Pathological complete response (pCR) was defined as the absence of viable tumor cells (ypT0N0M0) in the surgical resection specimen, and major pathologic response (MPR) was defined as less than 10% viable tumor remaining (21, 22). Additionally, the pathological response of the primary tumor was also assessed according to the College of American Pathologists (CAP) and National Comprehensive Cancer Network (NCCN) system (23) according to: tumor regression grade (TRG) 0 (no viable cancer cells), TRG 1 (single cells or rare small groups of cancer cells), TRG 2 (residual cancer with evident tumor regression), and TRG 3 (extensive residual cancer with no evident tumor regression). When there were disagreements between pathologists, a consensus would be reached through multi-head microscope review and discussion.

### Propensity score matching

Propensity score (PS) matching was conducted using logistic regression to create a PS for individual patients using demographic and clinical variables. The variables used to estimate the PS were age, gender, smoking status, FEV1%, tumor size, tumor location, pathologic type of tumor, clinical stage of tumor, and type of operation. The PS was calculated using a logistic model. The nearest neighbor matching was adopted with common caliper <0.1 and 1:1 matching. Each patient who underwent neoadjuvant immunotherapy combined with chemotherapy was matched with a patient who underwent neoadjuvant chemotherapy and had the closest PS.

### Statistical analysis

Data analysis was performed using SPSS 26.0 software (IBM SPSS Statistics, RRID: SCR_019096). Continuous data are presented as mean ± standard deviation (SD) and analyzed by the two-tailed t-test or rank sum test. Categorical data are presented as frequency and percentage (%) and were analyzed by either chi-square test or Fisher’s exact test. Survival curves were obtained using the Kaplan-Meier method, and the differences between survival curves were compared by the log-rank test. P<0.05 was considered significant. Multivariate Cox regression analysis was used to determine the risk factors for DFS, and to produce forest plot.

## Results

### Patients’ clinical characteristics and PS matching

A total of 62 cases were enrolled, including 20 patients (20/62, 32.26%) in the immunotherapy group and 42 patients (42/62, 67.74%) in the chemotherapy group. Baseline characteristics of all cases are presented in **Table 1**. The two groups were similar in terms of age, gender, smoking status, FEV1%, tumor size, tumor location, pathologic type of tumor, and clinical stage of tumor. In these two groups, the majority were male (85.00% and 85.71%), smokers (85.00% and 66.67%), squamous cell carcinoma (85.00% and 64.29%), and advanced stage cancer (65.00% and 57.14%).

**Table 1:** Patients’ baseline and clinical characteristics.

Characteristics	Unmatched patients			Matched patients^†^		
	Immunotherapy	Chemotherapy	P	Immunotherapy	Chemotherapy	P
**Patients(n)**	20	42		19	19	
**Age(years)**	58.05 ± 7.05	56.45 ± 8.66	0.475	58.58 ± 7.14	56.84 ± 8.90	0.511
**Gender (n [%])**			1.000			1.000
Male	17 (85.00%)	36 (85.71%)		16 (84.21%)	15 (78.95%)	
Female	3 (15.00%)	6 (14.29%)		3 (15.79%)	4 (21.05%)	
**Smoking status**			0.130			0.693
Absent	3 (15.00%)	14 (33.33%)		3 (15.79%)	5 (26.32%)	
Present	17(85.00%)	28 (66.67%)		16 (84.21%)	14 (73.68%)	
**FEV1 % predicted**	85.60 ± 16.00	78.58 ± 15.04	0.097	84.42 ± 15.52	81.14 ± 16.08	0.526
**Tumor size(cm)^‡^**	4.97 ± 2.10	4.67 ± 1.67	0.544	5.10 ± 2.08	4.92 ± 1.82	0.773
**Tumor location**			0.713			1.000
Right lobe	9 (45.00%)	21 (50.00%)		8(42.11%)	9 (47.37%)	
Left lobe	11 (55.00%)	21 (50.00%)		11 (57.89%)	10 (52.63%)	
**Tumor type**			0.093			0.660
Squamous cell	17 (85.00%)	27 (64.29%)		17 (89.47%)	15 (78.95%)	
Adenocarcinoma	3 (15.00%)	15 (35.71%)		2 (10.53%)	4 (21.05%)	
**Clinical stage^§^**			0.555			1.000
I-II	7(35.00%)	18 (42.86%)		7 (36.84%)	6 (31.58%)	
III	13 (65.00%)	24 (57.14%)		12 (63.16%)	13 (68.42%)	

^†^Patients with R1 or R2 resection were excluded When we carried out propensity score matching. ^‡^Tumor size (cm) prior to immunotherapy. ^§^Clinical stage prior to neoadjuvant therapy. Fisher's accurate test was adopted in Chi-square test after propensity score matching as the total number of samples was less than 40.

After excluding the patients with R1 or R2 resection and 1 to 1 propensity score matching, 19 pairs of patients were selected (**Table 1**). Also, the clinical characteristics including age, gender, smoking status, FEV1%, tumor size, tumor location, pathologic type of tumor, and clinical stage of tumor in the two groups were well balanced.

### Preoperative treatment and response to neoadjuvant therapy

**Figure 2:**
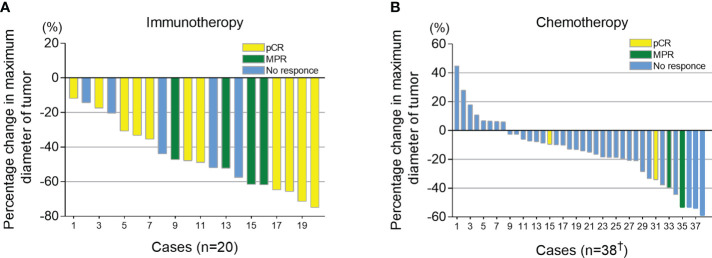
Imaging response (percentage change in maximum diameter of tumor) after neoadjuvant therapy. Combined with postoperative pathological results, patients who had a pathological complete response (pCR) were shown in yellow, major pathological response (MPR) shown in green and those with >10% viable tumor remaining are shown in blue. **(A)** Tumor size changes after neoadjuvant immunotherapy combined with chemotherapy. **(B)** Tumor size changes after neoadjuvant chemotherapy. ^†^, Of the 42 patients in chemotherapy group, 4 cases were not recorded due to the inadequate archived CT data.

All candidates received 2 cycles of neoadjuvant therapy. At 3-6 weeks after neoadjuvant therapy, iRECIST criteria were used to evaluate the imaging response of the tumor. Of the 42 patients in chemotherapy group, 4 cases were not recorded due to inadequate archived CT data. In the immunotherapy group, all patients had tumor regression (tumor regression ratio: 11.88%-75.00%) (**Figure 2A**). In the chemotherapy group, 30 patients had tumor regression (tumor regression ratio: 2.70%-58.97%) (**Figure 2B**).

### Surgical and postoperative results

The R0 removal rates for tumors were comparable between the immunotherapy group and chemotherapy group (19/20, 95.00% vs. 39/42, 92.86%, P=1.000) (**Table 2**). In the analysis of subsequent surgery and postoperative outcomes and propensity score matching, patients with R1 or R2 resection were excluded. The two groups were balanced in complete minimally invasive surgery, pneumonectomy, operative duration, blood loss, postoperative complications, and hospital stay.

**Table 2:** The comparison of the treatment results between the two groups.

Characteristics	Unmatched patients			Matched patients		
	Immunotherapy	Chemotherapy	P value	Immunotherapy	Chemotherapy	P value
**Patients(n)**	20	42		19	19	
**R0 resection**	19 (95.00%)^†^	39 (92.86%)^†^	1.000			
**Sleeve resection**	7 (36.84%)	4 (10.26%)	0.039	7 (36.84%)	3 (15.79%)	0.141
**pCR**	11 (57.89%)	2 (5.13%)	<0.001	11 (57.89%)	2 (10.53%)	0.002
**MPR**	15 (78.95%)	4 (10.26%)	<0.001	15 (78.95%)	4 (21.05%)	0.001
**TRG**			<0.001			0.001
0 - 1	15 (78.95%)	5 (12.82%)		15 (78.95%)	4 (21.05%)	
2 - 3	4 (21.05%)	34 (87.18%)		4 (21.05%)	15 (78.95%)	
**Minimally invasive surgery^‡^**	12 (63.16%)	16 (45.71%)	0.221	12 (63.16%)	9 (47.37%)	0.515
**Pneumonectomy**	2 (10.53%)	7 (20.00%)	0.610	2 (10.53%)	5 (26.32%)	0.405
**Operative duration (min)**	182.11 ± 67.19	170.31 ± 60.36	0.689	182.11 ± 67.19	179.63 ± 69.45	0.912
**Blood loss (ml)^§^**	120(20-1000)	150 (50-800)	0.403	120 (20-1000)	200 (50-800)	0.073
**Overall complications**	1 (5.26%)	6 (17.14%)	0.414	1 (5.26%)	3 (15.79%)	0.604
**Hospital stay (days)**	11.84 ± 4.17	14.36 ± 4.80	0.056	12.21 ± 4.37	14.89 ± 4.01	0.056

^†^Patients with R1 or R2 resection were excluded in the statistics of surgical outcomes in this table. ^‡^Minimally invasive surgery: Completely minimally invasive surgery. ^§^The data of blood loss was described with the median (range) as they were not normally distributed, and Mann-Whitney U rank sum test was used. TRG, tumor regression grade. pCR, Pathological complete response. MPR, Major pathological response, indicated that there was more than 10% viable tumor remaining in postoperative specimen. Sleeve resection includes bronchial sleeve and vascular sleeve resection.

The immunotherapy group had more sleeve resection (36.84% vs. 10.26%, p=0.039) including bronchial sleeve and vascular sleeve. The proportion of patients whose tumor reached pCR in the immunotherapy group was significantly higher than that in the chemotherapy group (57.89% vs. 5.13%, P<0.001) (**Table 2**). Additionally, the proportion of patients whose tumor reached MPR in immunotherapy group was significantly higher than that in chemotherapy group (78.95% vs. 10.26%, P<0.001). This difference persisted after propensity score matching (57.89% vs. 10.53%, P=0.002; 78.95% vs. 21.05%, P=0.001, respectively).

**Figure 3:**
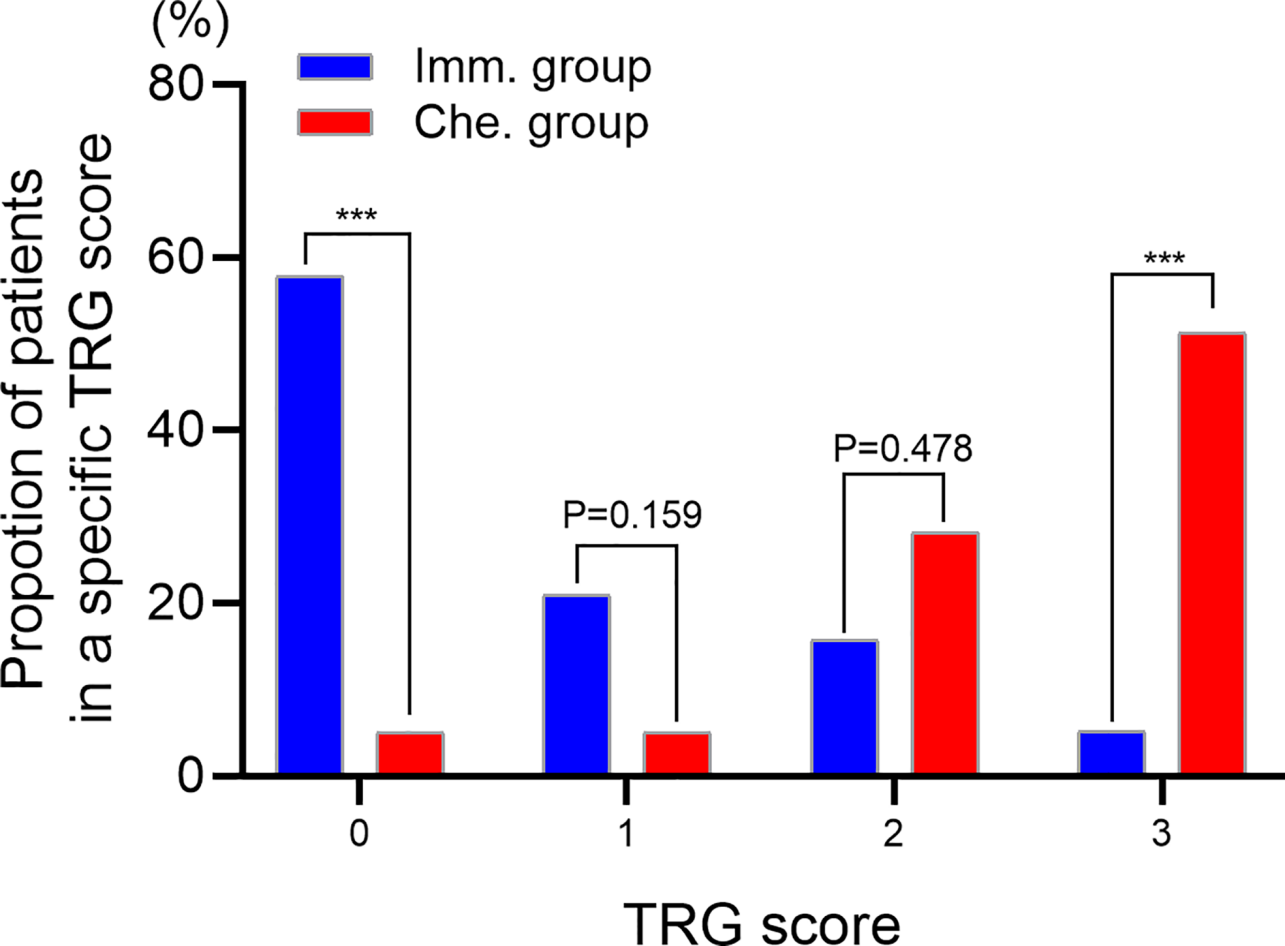
Evaluating the tumor regression grade (TRG) of postoperative specimens. TRG 0, no viable cancer cells. TRG 1, single cells or rare small groups of cancer cells. TRG 2, residual cancer with evident tumor regression. TRG 3, extensive residual cancer with no evident tumor regression. *** indicated P < 0.001. Imm., neoadjuvant immunotherapy combined chemotherapy. Che., neoadjuvant chemotherapy.

TRG scores of postoperative specimens were further analyzed (**Figure 3**). The results showed that the proportion of patients with a TRG score of “0” in the immunotherapy group was significantly higher than that in the chemotherapy group (57.89% vs. 5.13%, P<0.001), while the proportion with a TRG score of “3” was significantly lower than that in the chemotherapy group (5.26% vs. 51.28%, P<0.001).

### Survival analysis

**Figure 4:**
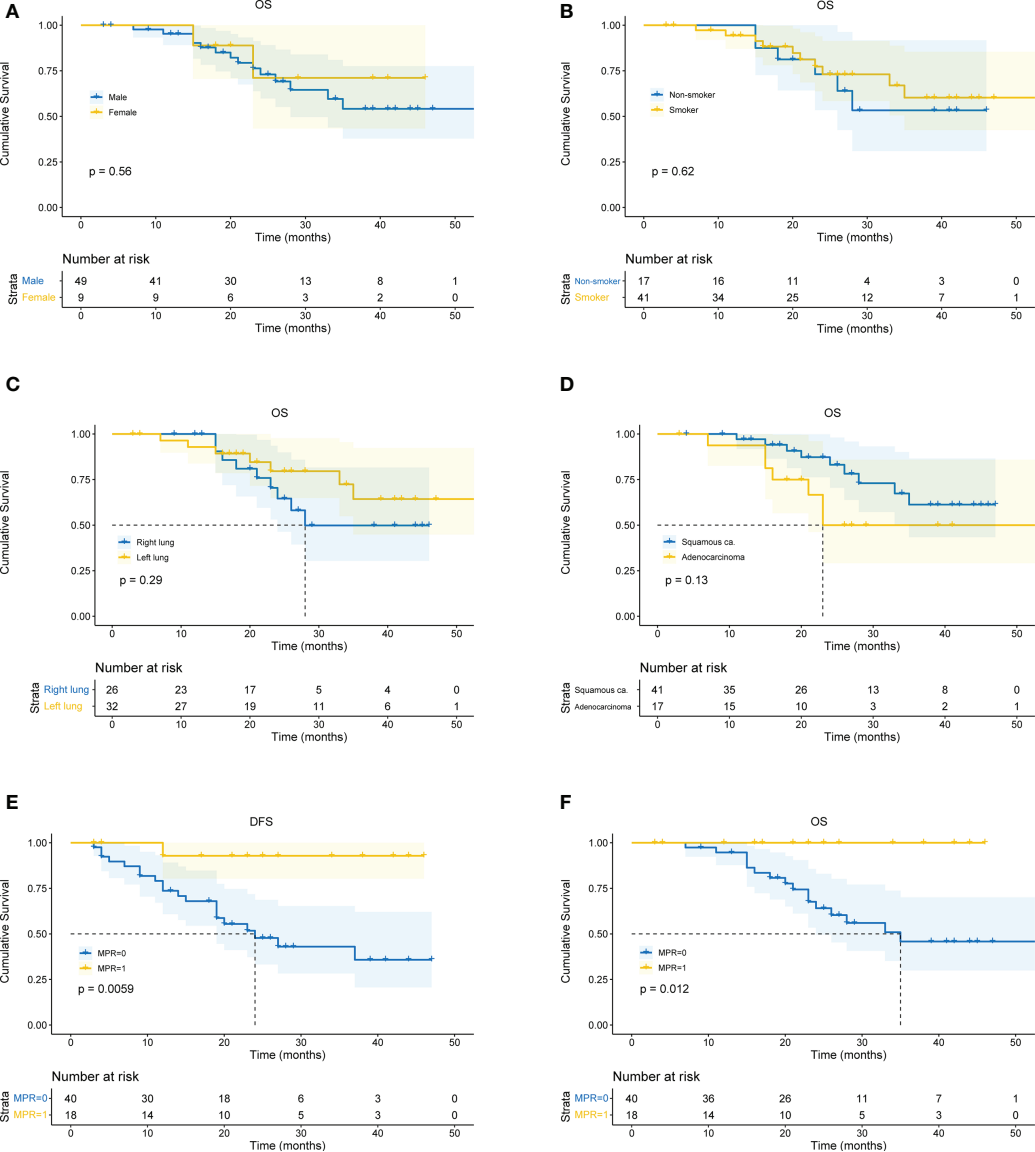
Kaplan-Meier curves for survival stratified by clinical parameters. **(A)** OS stratified by gender. **(B)** OS stratified by smoking status. **(C)** OS stratified by tumor location. **(D)** OS stratified by pathological type of cancer. **(E)** DFS stratified by MPR. **(F)** OS stratified by MPR. OS, overall survival. DFS, disease free survival. MPR, major pathological remissions. ca., carcinoma.

All patients received CT and PET-CT evaluation on follow-up. The last follow-up was in June 2022. For all patients, the overall median follow-up period was 24 months (4 – 59 months). The 1-year OS was 93.1%, 2-year OS was 72.1%, and 3-year OS was 59.8%. There was no difference in survival curves between male and female, smoker and non-smoker, squamous cell carcinoma and adenocarcinoma, or right lung cancer and left lung cancer (**Figures 4A-D**). Moreover, patients who achieved MPR (including pCR) had significantly better OS (P=0.018) and DFS (P=0.016) (**Figures 4E, F**).

**Figure 5:**
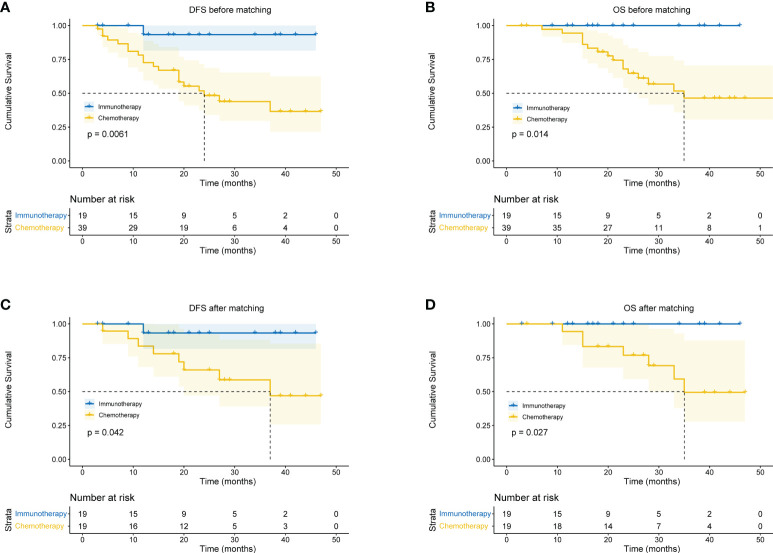
Kaplan-Meier curves for survival stratified by neoadjuvant therapy before or after propensity score matching. **(A)** DFS before PS matching. **(B)** OS before PS matching. **(C)** DFS after PS matching. **(D)** OS after PS matching. OS, overall survival; DFS, disease free survival; PS, propensity score.

**Figure 6:**
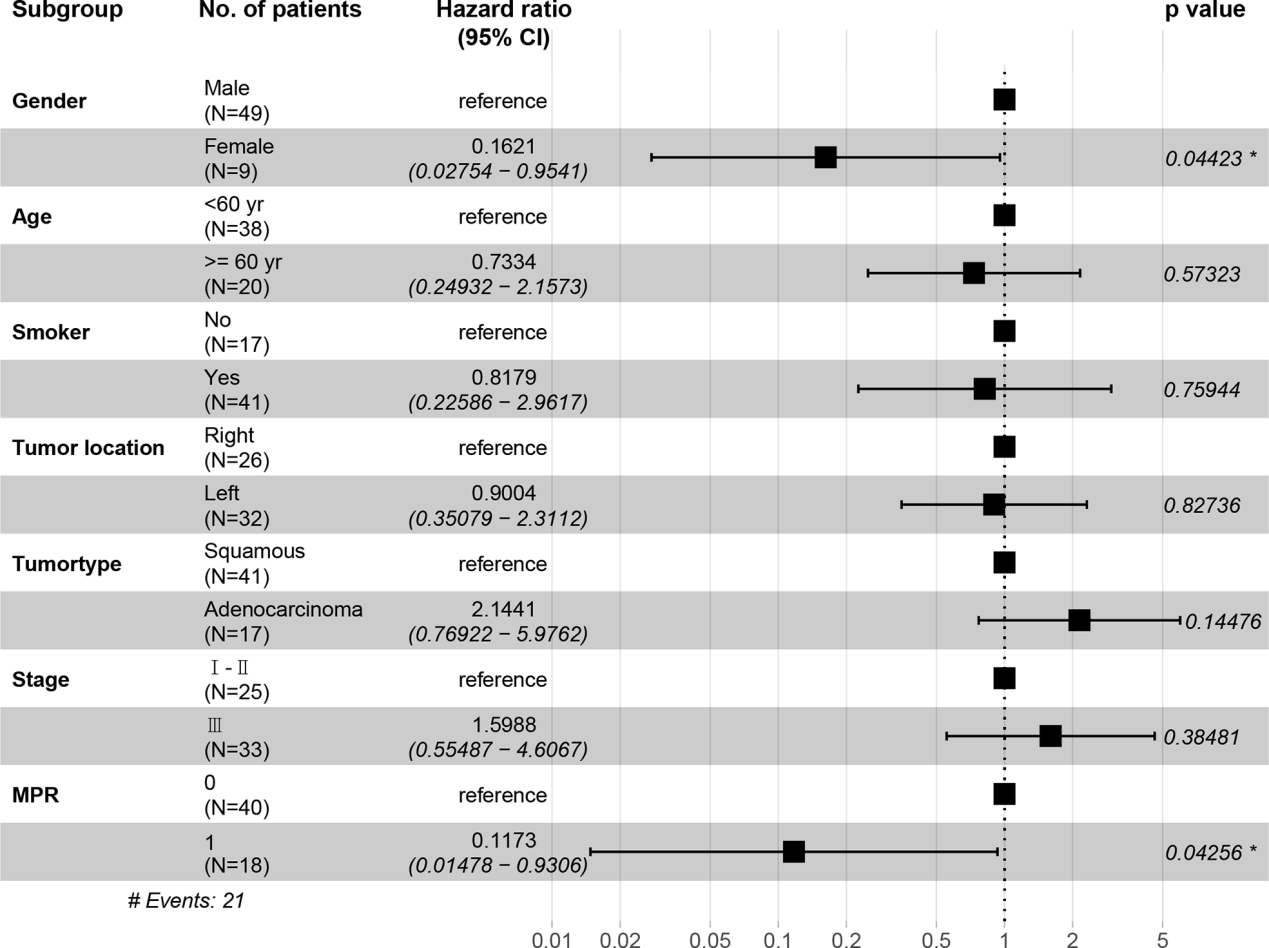
The hazard ratio of DFS among each subgroup shown in the forest plot. CI, confidence interval. *, indicates a significant difference. Stage, indicates the tumor stage at baseline.

In the immunotherapy group, no death event occurred and 1 case experienced postoperative recurrence. The median follow-up period was 19 months (6 - 46 months), and the 3-year OS was 100%. In the chemotherapy group, the median follow-up period was 25 months (4 - 59 months). The 1-year OS was 77.3%, 2-year OS was 64.0%, and 3-year OS was 49.6%. Patients in the immunotherapy group had significantly better OS than those in the chemotherapy group (P = 0.014), and longer DFS (P = 0.006 (**Figures 5A, B**). After propensity score matching, we re-evaluated the impact of the two neoadjuvant therapies on the prognosis of patients. Neoadjuvant immunotherapy combined with chemotherapy was significantly associated with better OS (P=0.027) and better DFS (P < 0.042) (**Figures 5C, D**). Through COX regression analysis, female with hazard ratio 0.16 (95% CI, 0.03 to 0.95) and achieving MPR with hazard ratio 0.12 (95% CI, 0.01 to 0.93) were protective factors for DFS (**Figure 6**).

## Discussion

We retrospectively analyzed the therapeutic outcomes and prognosis of neoadjuvant immunotherapy combined with chemotherapy for resectable NSCLC compared with neoadjuvant chemotherapy. Our results showed that neoadjuvant immunotherapy combined with chemotherapy can significantly improve the imagological regression of tumors, and patients had a significantly higher pCR rate, MPR rate, and better long-term prognosis with this combined therapy.

In our study, neoadjuvant immunotherapy combined with chemotherapy did not increase the risk of delayed surgery. It is well known that early diagnosis and timely operation can significantly improve the cure rate and survival of lung cancer (24). Tang et al. pointed out that when the time between diagnosis of lung cancer and surgery was greater than 50 days, patients’ 1- and 5-year survival rates decreased (25). A study by Meyer et al. demonstrated that delaying surgery in favor of neoadjuvant therapy did not impair quality of life or result in additional tumors in cancer patients (26). In our study, we found that no patients in the neoadjuvant immunotherapy group experienced metastasis, recurrence, or death and that the neoadjuvant immunotherapy group experienced significantly improved DFS and OS compared with the neoadjuvant chemotherapy group.

Neoadjuvant immunotherapy combined with chemotherapy will not increase the unresectable rate. In this study, the R0 resection rate in the neoadjuvant immunotherapy group was higher than in the neoadjuvant chemotherapy group (19/20, 95.00% vs 39/42, 92.86%, *p*=1.000). In CheckMate 816 trial, the R0 rate in neoadjuvant immunotherapy group was 83% (27). We adequately considered the integrated and radical resection of tumor. Not only did we ensure R0 resection rate, but the extent of resection was accorded to that before neoadjuvant therapy. This may also have something to do with our strict case selection. Additionally, no post-treatment tumor progression was observed in the neoadjuvant immunotherapy group (there were no increases in tumor diameter, no increases in tumor invasion, and no tumor metastasis). Of the patients in this group, there was 1 case of R2 resection, which was unresectable due to the invasion of the main bronchus by the seventh group of lymph nodes. After neoadjuvant therapy, the tumor diameter decreased by 44%, and the seventh group of lymph nodes decreased by 20%. In the neoadjuvant chemotherapy group, there were 4 cases without R0 resection. Therefore, the aim of neoadjuvant therapy in patients in more advanced stages is to shrink the tumor and convert tumors with unresectable margins into resectable lesions (28).

However, in some cases, the complexities of surgery could increase even after neoadjuvant chemoradiotherapy decreased the tumor stage (29, 30). This could be because neoadjuvant therapy can cause local tissue adhesions, which were observed in patients experiencing significant responses to treatment. In this study, there was no significant difference observed in the surgery-related data between the two groups. The operation time for the neoadjuvant immunotherapy group was slightly longer than for the neoadjuvant chemotherapy group, which could be due to: 1) the small number of patients included in the study (which could create bias); and 2) killing tumor cells with PD-1/PD-L1 inhibitor requires antigen presentation by the tumor cells, which can then be recognized by the host T-cells. The activated T-cells can release cytokines after the blockade of the immunosuppressive PD1/PD-L1 interaction by inhibitory antibodies, which can kill the tumor cells (31). After killing the tumor cells, the local tumor bed and surrounding tissue are replaced by fibrous tissue, forming denser adhesions, which increases the difficulty of surgery and prolong the operation time.

The lack of a standardized approach for reporting the pathology of lung cancer patients resected after neoadjuvant therapy could indicate that pathologists are not involved in study designs (21, 32). We used the newer CAP/NCCN guidelines for tumor regression grading following neoadjuvant chemotherapy to evaluate postoperative pathological specimens in all cases, which increased the reliability of the study (33, 34). Our results demonstrate that the tumor regression grade of the neoadjuvant immunotherapy group was significantly better than that of the neoadjuvant chemotherapy group.

Neoadjuvant immunotherapy combined with chemotherapy significantly increased rates of pCR and MPR. One major limitation is MPR’s lack of precision due to inherent inter-observer variability (13). However, it is well known that pCR or MPR is the primary endpoint of many neoadjuvant immunotherapy studies (22) and is associated with favorable tumor prognosis and improvements in overall survival (35, 36). MPR has been identified as a surrogate endpoint for survival in patients who received neoadjuvant therapy prior to lung cancer resection, while MPR improved the five-year overall survival rate from 40% to 85% (37, 38). Since MPR is associated with improved survival rates, it could provide a faster way to compare different neoadjuvant treatment options and reduce the time required to evaluate neoadjuvant therapies. Compared with patients who received only preoperative chemotherapy, the MPR rate was higher (16%) in patients with immunotherapy and chemotherapy (39) which was consistent with our results (40). However, it was difficult to obtain accurate postoperative pathological staging. Therefore, the comparison that based on the clinical TNM stage may be bias and we found that there were no significant differences between clinical stage I-II and stage III for DFS and OS respectively. Similarly, female seem to be a protective factor for DFS, possibly because of the small sample size and few events of DFS.To assess whether an increase in the pCR or MPR rate produced a survival benefit, we further analyzed OS and DFS. Survival rates were significantly higher in patients with pCR or MPR than in patients without pCR or MPR. Neoadjuvant immunotherapy significantly improved the survival rate of patients compared with the neoadjuvant chemotherapy group. Additionally, studies have demonstrated that neoadjuvant immunotherapy increased tumor-specific CD8+T cells in peripheral blood and organs. Therefore, neoadjuvant immunotherapy is better able to eradicate distant metastases and increase long-term survival rates after primary tumor resection than adjuvant immunotherapy (28). Currently, many clinical trials have analyzed the survival rates of lung cancer patients with neoadjuvant immunotherapy, and more attention is being paid to the safety and feasibility of neoadjuvant therapies. Most of the selected primary endpoints are either MPR or pCR (15, 41), or a short-term survival analysis (1-2 years) (42–44). In our study, the follow-up period was 24 months (6-53 months). There were no deaths and only 1 case of recurrence in the neoadjuvant immunotherapy group, which was an encouraging result.

Besides, our study provided clinical experiences in refining specific applications. Adjustments of clinical workflow and careful consideration for patient selection are undoubtedly necessary for neoadjuvant and adjuvant immunotherapy.

However, there were some limitations and shortcomings in our study. First, it was retrospective and lacked sufficient statistical analysis. Second, the sample size recruited was small and the follow-up time was short. We will continue this work with further follow-up and hope to obtain more accurate results in future studies.

## Conclusions

The combination of neoadjuvant immunotherapy and chemotherapy can effectively shrink tumors, improve pCR and MPR rates of tumors, and help patients achieve better OS and DFS.

## Author’s note

*Reporting Checklist:* The authors have completed the STROBE reporting checklist.

## Data availability statement

The original contributions presented in the study are included in the article/supplementary material. Further inquiries can be directed to the corresponding author.

## Ethics statement

The studies involving human participants were reviewed and approved by Ethics Committee of Army Medical Center of PLA, approval number: 2021-273. The patients' individual consent for this retrospective analysis was waived.

## Author contributions

Conception and design: WG. Administrative support: QT, RW. Provision of study materials or patients: CS, JZ, HN, QT, RW, WG. Collection and assembly of data: FD, XWa, YW, KL. Data analysis and interpretation: FD, XWu. Manuscript writing: All authors. Final approval of manuscript: All authors contributed to the article and approved the submitted version.

## Conflict of interest

The authors declare that the research was conducted in the absence of any commercial or financial relationships that could be construed as a potential conflict of interest.

## Publisher’s note

All claims expressed in this article are solely those of the authors and do not necessarily represent those of their affiliated organizations, or those of the publisher, the editors and the reviewers. Any product that may be evaluated in this article, or claim that may be made by its manufacturer, is not guaranteed or endorsed by the publisher.
